# Behavioural Adaptation to Hereditary Macular Dystrophy: A Systematic Review on the Effect of Early Onset Central Field Loss on Peripheral Visual Abilities

**DOI:** 10.22599/bioj.177

**Published:** 2021-06-16

**Authors:** Aishah Baig, David Buckley, Charlotte Codina

**Affiliations:** 1 University of Sheffield, GB

**Keywords:** macular dystrophy, peripheral vision, cortical plasticity, perceptual learning

## Abstract

**Purpose:**

Hereditary macular dystrophies (HMD) result in early onset central field loss. Evidence for cortical plasticity has been found in HMD, which may enhance peripheral visual abilities to meet the increased demands and reliance on the peripheral field, as has been found in congenitally deaf adults and habitual action video-game players. This is a qualitative synthesis of the literature on the effect of early onset central field loss on peripheral visual abilities. The knowledge gained may help in developing rehabilitative strategies that enable optimisation of remaining peripheral vision.

**Methods:**

A systematic search performed on the Web of Science and PubMED databases yielded 728 records published between 1809 to 2020, of which seven case-control studies were eligible for qualitative synthesis.

**Results:**

The search highlighted an overall paucity of literature, which lacked validity due to small heterogeneous samples and deficiencies in reporting of methods and population characteristics. A range of peripheral visual abilities at different eccentricities were studied. Superior performance of HMD observers in the peripheral field or similarities between the preferred retinal loci (PRL) and normal fovea were observed in four of seven studies. Findings were often based on studies including a single observer. Further larger rigorous studies are required in this area.

**Conclusions:**

Spontaneous perceptual learning through reliance on and repeated use of the peripheral field and PRL may result in some specific superior peripheral visual abilities. However, worse performance in some tasks could reflect unexpected rod disease, lack of intensive training, or persistent limitations due to the need for cones for specific tasks. Perceptual learning through training regimes could enable patients to optimise use of the PRL and remaining peripheral vision. However, further studies are needed to design optimal training regimes.

## Introduction

Hereditary macular dystrophies (HMD) are inherited congenital or juvenile-onset macular dystrophies, which affect the functioning of cone photoreceptors. Cones are most densely situated at the fovea in the macula at 0° retinal eccentricity (Curcio et al. 1991). Cone density rapidly declines with increasing retinal eccentricity and by 1.75°, approaching the limit of the fovea (Engbert et al. 2002), cone density is approximately halved (Curcio et al. 1991). Hereditary macular dystrophies therefore result in varying degrees of bilateral central field loss from an early age (Altschwager et al. 2017; Pascual-Camps et al. 2018). Affected individuals are left with their peripheral visual field to rely on for visual functions, including those requiring the fine discrimination abilities of central vision, which the peripheral retina is not designed to accommodate (Boucart et al. 2010; 2013). This primarily affects the ability to read and recognise faces, reducing the quality of life of populations with HMD (Miedziak et al. 2000; Szlyk et al. 1998).

In order to optimise use of the peripheral visual field for such functions, many individuals spontaneously adopt specific points of peripheral retina to fixate objects of interest, much like the fovea would (Cheung & Legge 2005; Cummings et al. 1985; Fletcher & Schuchard 1997). These points are now referred to as preferred retinal loci (PRL) (Cummings et al. 1985; Fletcher & Schuchard 1997). The development of PRL provides evidence for cortical plasticity in HMD. Through experience and behaviour altering sensory input and neuronal connections, cortical plasticity can help compensate for impaired functions and adapt to changing demands in our environment even in adulthood (Gilbert et al. 2001; Konorski 1948; Mateos-Aparicio & Rodriguez-Moreno 2019; Pascual-Leone et al. 2005).

There may also be other behavioural adaptations in response to increased demands on the peripheral field, such as enhanced detection, localisation, discrimination of static and moving stimuli in the peripheral field or visual field expansion, as has been observed in the congenitally deaf (Bottari et al. 2010; 2011; Buckley et al. 2010; Codina et al. 2017; Proksch & Bavelier 2002; Shiell, Champoux & Zatorre 2014; Stevens & Neville 2006) and habitual action video game players (Bavelier et al. 2012a; Green & Bavelier 2003; 2006; 2007; Buckley et al. 2010; Feng et al. 2007; Mishra et al. 2011; Wu et al. 2012; Wu & Spence 2013).

The neural basis for these enhancements may lie in the reorganisation of the cortical architecture of primary visual cortex/V1; increasing the number of neurons available to process peripheral stimuli (Baker et al. 2005; Levine et al. 2015; Liu et al. 2010; Sabbah et al. 2017). This is known as cortical reorganisation. On the other hand, functional reorganisation may take place, where the normal cortical structure is retained but pre-existing neuronal networks are unmasked or strengthened that would otherwise be suppressed in those with normal sight (Baker et al. 2005; 2008; Baseler et al. 2011; Masuda et al. 2008; 2010; Morland 2015).

Similarly to action video-game players and the congenitally deaf, there is evidence to support cortical reorganisation of V1 in congenital cone dystrophies, such as achromatopsia (Morland 2001; Baseler et al. 2002) and functional reorganisation in juvenile-onset macular dystrophies (Baker et al. 2005; 2008; 2010; Baseler et al. 2011; Lorenz et al. 2015; Masuda et al. 2008; Morland 2015; Plank et al. 2017; Sanda et al. 2018; Sabbah et al. 2017; Liu et al. 2010), which may also lead to enhanced peripheral visual abilities.

It has been suggested that, through functional reorganisation, superior performance in action video-game players in particular is achieved by better distribution of attentional resources to task-relevant stimuli across the visual field (Bavelier et al. 2012a; 2012b; Bediou et al. 2018; Green & Bavelier 2003; 2006; 2012; Green, Li & Bavelier 2010; Wu et al. 2012; Wu & Spence 2013).

Perceptual learning has also been implicated in the explanation for superior visual performance in action video-game players (Bavelier et al. 2012b; Green, Li & Bavelier 2010). Perceptual learning is an example of experience-dependent cortical plasticity, where the adult brain can improve long-term visual performance through repeated visual experience (Ahissar & Hochstein 1997; Fahle & Poggio 2002; Gibson 1963; Gibson 1969; Gilbert et al. 2001; Watanabe, Nanez & Sasaki 2001; Watanabe & Sasaki 2015). This may be achieved by altering attentional control through functional reorganisation (Chen et al. 2016; McAdams & Maunsell 1999a; 1999b; Mukai et al. 2007; Schafer, Vasilaki & Senn 2007). With repeated use of a PRL, perceptual learning may direct more attentional resources to this location, in order to enhance its use (Dilks et al. 2009; Liu et al. 2010; Masuda et al. 2008; Plank et al. 2013; 2017).

This systematic review is a qualitative synthesis of the evidence on the effect of early-onset central field loss on peripheral visual abilities. This review aims to facilitate a better understanding of the integrity of the peripheral field in and outside the PRL of those with HMD. The knowledge gained may help develop rehabilitative strategies which optimise use of healthy peripheral retina.

## Methods

The search strategy followed that outlined by Bettany-Saltikov (2012). Initially, the research question was separated into component parts: the population, exposure, and outcome. The population being studied were patients with HMD, their exposure was to early onset central field loss and the outcome being reviewed was their peripheral visual abilities. Keywords for each component part of the research question were identified, along with their synonyms, truncations, and abbreviations. The words identified generated search terms, which were then combined using Boolean operators to formulate a ‘search strategy string’, as shown in ***Table 1***. Right-hand truncations are accompanied by the ‘*’ symbol. Within each component part, search terms were combined with ‘OR’, whilst ‘AND’ was used to combine all search terms between component parts.

**Table 1:** Search strategy string combining search terms from each component part of the research question with Boolean operators.


SEARCH TERMS		

POPULATION	EXPOSURE	OUTCOME

1 ‘achromatopsia’ 2 ‘achromat*’ 3 ‘stargardt*’ 4 ‘maculopath*’ 5 ‘macular dystroph*’ 6 ‘best disease’ 7 ‘best’s disease’ 8 ‘vitelliform’ 9 ‘cone dystroph*’ 10 ‘macular degeneration’ 11 ‘macular lesion*’ 12 ‘central retinal lesion*’	14 ‘CFL’ 15 ‘central field defect*’ 16 ‘central visual field defect*’ 17 ‘central visual field loss’ 18 ‘central scotoma*’ 19 ‘central visual field scotoma*’ 20 ‘central vision loss’	22 ‘peripher*’ 23 ‘visuospatial’ 24 ‘spatial’ 25 ‘attention’ 26 ‘plasticity’ 27 ‘neuroplasticity’ 28 ‘neuro-plasticity’ 29 ‘reorganisation’ 30 ‘re-organisation’ 31 ‘reorganization’ 32 ‘re-organization’ 33 ‘remapping’ 34 ‘re-mapping’ 35 ‘adapt*’ 36 ‘compensat*’ 37 ‘cortical magnification’ 38 ‘magnification factor’ 39 ‘eccentric*’ 40 ‘pseudofovea*’ 41 ‘pseudo-fovea*’ 42 ‘parafovea*’ 43 ‘para-fovea*’ 44 ‘preferred retinal locus’ 45 ‘PRL’ 46 ‘paramacular’ 47 ‘para-macular’ 48 ‘paracentral’ 49 ‘para-central’ 50 ‘extra-fovea*’ 51 ‘extrafovea*’

13 Combine terms 1 to 12 using ‘OR’	21 Combine terms 14 to 20 using ‘OR’	52 Combine terms 22 to 51 using ‘OR’

Combine 13, 21 and 52 using ‘AND’		


The string was inputted into Web of Science (1864 to 06/2020) and PubMed (1809 to 06/2020). Reference lists of primary literature, books, review articles and grey literature identified from the database searches were also examined for further relevant records.

Predetermined inclusion and exclusion criteria were applied to the search results: Firstly, studies were excluded if they included populations who had HMD and concomitant ocular disease, which may confound results. If populations with other diagnoses were included within the same study, results for patients with HMD must have been analysed separately.

Only studies including patients above the age of 10 were eligible, to ensure performance on peripheral visual tasks was not affected by level of understanding or cooperation. By age 10, static perimetry has been found to be similar to adult levels (Patel et al. 2015). Limiting inclusion of studies to those with age-matched samples would have been preferable; however, it was not possible due to the lack of literature available.

Males have been found to perform better in spatial learning and navigation (Driscoll et al. 2005; Iachini et al. 2005) and on a peripheral visual attention task (Feng et al. 2007). Therefore, restricting studies to those with gender-matched samples may have also been beneficial. However, scarcity of available literature would not allow for this.

Small heterogeneous sample sizes are consistently observed in the literature on HMD (Castaldi et al. 2020). Therefore, eligibility criteria surrounding the stage and severity of disease were kept to a minimum to allow for the collection of adequate data. However, it was crucial that patients had HMD with bilateral foveal-involving central scotomas. This could be stated explicitly or demonstrated through poor visual acuities and presence of a PRL for example. This criterion was set to ensure sufficient central visual impairment to elicit changes in peripheral visual abilities, should they exist. Studies on cortical reorganisation suggest that unilateral, bilateral non-absolute or foveal-sparing central scotomas do not produce changes in cortical processing of peripheral stimuli (Baker et al. 2008; Dilks et al. 2014; Masuda et al. 2008). Therefore, it was expected that similar scotomas may not produce changes to peripheral visual abilities.

Although it would make direct comparison between studies difficult, studies including a measurement of any visual ability at any eccentricity beyond the fovea (central 2°) (Engbert et al. 2002) were included in this review, as literature was known to be sparse. These were referred to as peripheral visual abilities for the purpose of this review.

This review was limited to human quantitative studies. Interventional studies were excluded as they were not appropriate to assess the baseline peripheral visual abilities of patients with HMD.

The following data was extracted from each study: study purpose, study design and outcome measurements, population and sampling, analysis and results, and conclusions. The Critical Review Form for Quantitative Studies from the McMaster University Occupational Therapy Evidence-Based Practice Research Group was then used to critically appraise the quality of the studies.

## Results

### Search results

**Figure 1:**
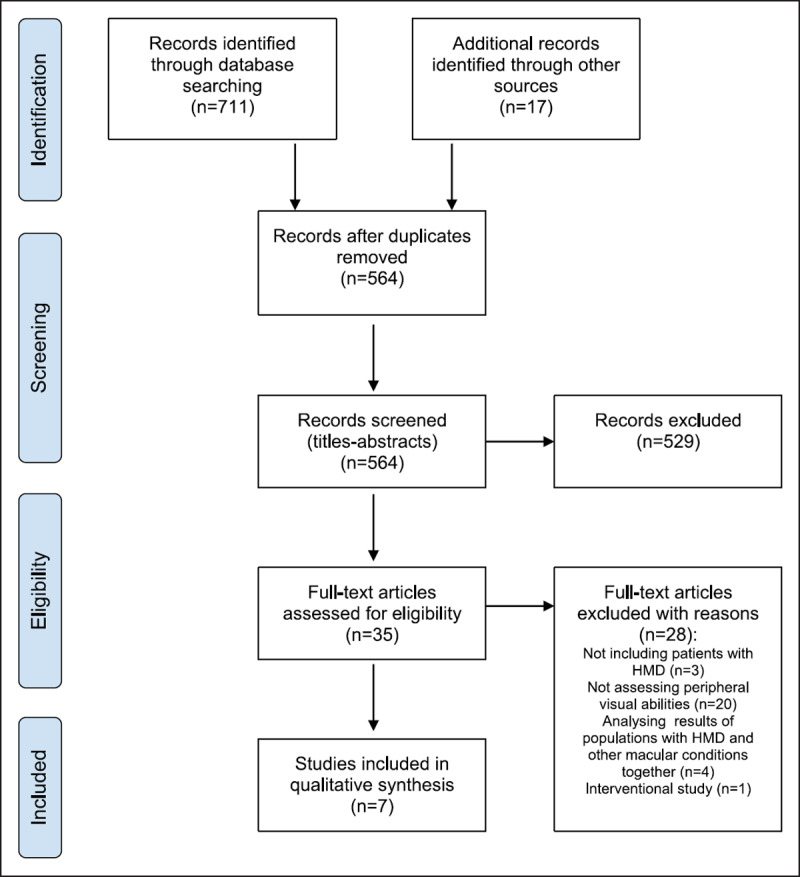
Study selection process for this systematic review.

The Web of Science and PubMed searches yielded 479 and 232 records, respectively. Collectively, database search results and those from other sources, identified 728 records. Titles of all records were read to exclude duplicates, which resulted in 564 remaining records for screening. The titles and abstracts of the remaining records were screened to determine if they met the exclusion criteria. This resulted in 529 records being excluded. The final 35 primary study records were read fully to determine eligibility and it was found that seven records met all the inclusion criteria for qualitative synthesis. No non-English studies were identified to be read fully. ***Figure 1*** illustrates how the primary studies were selected for review from the collective search results, including reasons for exclusion of fully read studies.

See Appendix 1 for a summary of the seven studies included for qualitative synthesis, including the study design, sample, measurement(s), and main findings/conclusions.

### Study purpose

A range of different peripheral visual abilities were investigated in populations with HMD, as shown in Appendix 1. Two of the studies investigated visual abilities at the PRL alone (Chung 2013; Mei & Leat 2007a), whilst the remaining studies investigated visual abilities outside of the PRL. Six studies compared peripheral visual function of both cases and controls, whilst one study compared PRL performance in cases to foveal performance in controls (Mei & Leat 2007a). Due to the variety of methods, direct comparison between studies was not always possible or appropriate.

### Study design and outcome measurements

All seven studies identified were case-control studies, which were the most appropriate study design to explore differences in the baseline peripheral visual abilities between those with and without HMD.

Not only were different outcome measurements taken across the studies, but these were taken at different retinal eccentricities. Therefore, even for those studies that investigated the same peripheral visual ability, results did not always allow for direct comparison.

Four studies measured the contrast sensitivity function at different eccentricities and luminance levels. Three of these studies used a two-alternative forced-choice staircase procedure, which is popular for measuring detection thresholds such as contrast sensitivity, as they are efficient and minimise bias, increasing validity (Fechner 1860; Garcia-Perez 1998; Green & Sweets 1966; Pelli & Bex 2013). Burton et al. (2016) used a two-alternative forced-choice procedure, but it was unclear if a staircase procedure was also used.

A two-alternative forced-choice procedure was also used for all other detection threshold tasks of the seven studies. Discrimination and recognition tasks on the other hand required the participant to make a judgement on one stimulus rather than two, which introduced individual biases.

Six of the studies randomised and counterbalanced stimuli and/or conditions to reduce order effects causing habituation and anticipation of stimuli. Chung (2013) also confirmed test-retest reliability at up to two more time points in a few observers. Time points were not specified, however. Other studies used repeated trials, but only in one sitting, or it was not stated clearly if multiple time points were used. The time interval between retests for reliability is crucial, as a greater time interval reduces the likelihood of learning or carry-over effects, which invalidate the results of the retest for reliability (Allen & Yen 1979). Hess, Nordby, and Pointer (1987) did not clearly state the use of repeated trials, randomisation or counterbalancing of conditions, reducing the reliability and validity of results.

The ability to monitor participants’ gaze and viewing patterns during trials is critical in the designs of these studies, to ensure results reflect measurements in the correct part of the visual field. Studies used video recordings (Chung 2013) and infrared eye tracking systems (Boucart et al. 2010; Nugent et al. 2003), to be reviewed after trials. Trials were then discarded if significant eye movements were detected. It appeared that Casco et al. (2003) monitored gaze manually in real-time. This method may be prone to error if the observer missed a significant eye movement, which then cannot be rechecked in the absence of a recording. Hess, Nordby, and Pointer (1987), Mei and Leat (2007), and Burton et al. (2016) did not provide any information on their method of monitoring gaze during trials, reducing the validity of results.

### Population and sampling

Diagnoses of participants included: achromatopsia (Burton et al. 2016; Hess, Nordby & Pointer 1987), Stargardt’s macular dystrophy (Boucart et al. 2010; Casco et al. 2003; Chung 2013), and other unspecified juvenile-onset macular dystrophies (Mei & Leat 2007a; Nugent et al. 2003), which are all types of HMD resulting in early onset central field loss.

There were large inconsistencies in reporting of essential visual parameters of participants, such as visual acuity, scotoma size, PRL location and eccentricity, fixation stability, and duration of central field loss. Consistency in reporting would have been particularly helpful in determining the generalisability of findings and if severity of disease correlated with findings.

Case sample sizes were small overall; two studies had only one participant in their case group (Casco et al. 2003; Hess, Nordby & Pointer 1987), and the largest sample consisted of 11 (Burton et al. 2016). There was also a level of heterogeneity within and between study samples. Small heterogenous samples would be expected when studying these rare and variable conditions and are therefore representative of the population. However, heterogeneity in such small samples leads to a reduction in power and ability to generalise and draw conclusions from results (Castaldi, Lunghi & Morrone 2020), along with studies including only one case participant. All studies had larger control to case sample sizes likely to increase statistical power with limited case populations (Boucart et al. 2010; Fleiss, Levin & Paikl 2003; Gail et al. 1976).

Only one of the studies explicitly stated that their groups were age-matched (Casco et al. 2003). Two more of the studies did not explicitly state that the groups were age-matched but had similar ages between groups (Boucart et al. 2010; Burton et al. 2016).

Three of the studies did not have similar ages between groups: The ages of the control group in the study by Chung (2013) investigating crowding were older on average. However, age has not been found to affect peripheral crowding (Astle et al. 2014; Malavita, Vidyasagar & McKendrick 2017), therefore age differences may not have affected results here. Mei and Leat (2007a) also had an older control group in their study on suprathreshold contrast matching. The authors did, however, reference another of their works of the same year, which demonstrated no effect of age on suprathreshold contrast matching (Mei & Leat 2007b). Nugent et al. (2003) used a younger control group. Foveal contour integration has been found to be worse with age (Roudaia, Bennett & Sekuler 2013), but to the author’s knowledge, the effect of age on peripheral contour integration has not been studied.

Only two studies reported the gender of all participants, and these were similar between groups (Burton et al. 2016; Chung 2013). But no studies reported that samples were gender-matched.

Very few studies have investigated gender differences in peripheral visual abilities. Feng, Spence and Pratt (2007), however, found that males performed better on a peripheral visual attention task. Of the studies investigating foveal abilities, there are mixed results, such as those for contrast sensitivity (Abramov et al. 2012; Brabyn & McGuinness 1979; Solberg & Brown 2002). This is likely due to small sample sizes and varied methods (Shaqiri et al. 2018). A large study investigating 15 different visual tasks found that males significantly outperformed females on six of the tasks, including a biological motion task, with a medium effect size. Due to the difference in performance of 6/15 tasks, these authors advised controlling for gender in research on all visual tasks (Shaqiri et al. 2018).

None of the studies reported an established reliable method of recruiting cases or control participants, therefore the study samples may be prone to selection bias and misrepresentation (Gail et al. 2019). In general, due to the non-randomised nature of the study design, selection bias is not uncommon for case-control studies (Schlesselman & Stolley 1982). Five studies did not provide details of recruitment methods, two of which included authors as participants introducing bias due to their knowledge of the field (Hess et al. 1987; Nugent et al. 2003). In the study by Boucart et al. (2010), controls were ophthalmology medical staff or university students who may also have knowledge of the research methods creating bias.

### Analysis and results

Contrast sensitivity was assessed in four studies. Contrast sensitivity refers to how able the visual system is at distinguishing objects from other objects and their background due to differing levels of light and dark (Pelli & Bex 2013). Regardless of retinal eccentricity, three of four studies that compared peripheral contrast sensitivity in HMD to controls found significant impairments in HMD in photopic/bright light conditions, but much less so for lower spatial frequencies, which correspond to coarse rather than finer features (Casco et al. 2003; Mei & Leat 2007a) or in scotopic/low light conditions (Hess, Nordby & Pointer 1987). In fact, Casco et al. (2003) found no statistically significant difference between their patient with Stargardt’s and controls for low spatial frequencies. Results were reported in terms of z scores, with 1.65 indicating statistical significance. All z-scores were below –1.0 for lower spatial frequencies. Under scotopic conditions, Hess, Nordby, and Pointer (1987) also found equivalent contrast sensitivity to controls across all spatial frequencies in their single achromat observer. However, these authors did not perform tests for statistical significance, which may reflect the small sample size of three participants and the year in which the study was published, as reporting of statistical significance was less prevalent (Altman 1998). Burton et al. (2016) only found that three of nine of their achromats demonstrated results in keeping with the pattern above. Lack of results from two participants due to availability may not have altered overall findings as even if both had also shown results similar to the above, this would still be less than half of their sample. Burton et al. (2016) did not report the statistical significance of contrast sensitivity functions to comment on.

The larger two of these four studies demonstrated considerable variability in contrast sensitivity functions of HMD (Burton et al. 2016; Mei & Leat 2007a). Possible reasons for this are unclear. Only the effect of age and genotype were explored by Burton et al. (2016) and were not found to be correlated with findings. Differences in severity of disease between participants may have been a factor. Burton et al. (2016) only recorded visual acuity of observers, but there was no correlation with this and performance. Mei and Leat (2007a) did not record any visual characteristics of patients for further analysis.

On a contrast-matching task comparing the ability of the normal fovea and the PRL to match the contrast of one test grating to another, significant mixed analysis of variance interactions between group, contrast levels and spatial frequency revealed significance of p < 0.001, indicating that compared to the normal peripheral retina of controls, for low and medium contrast levels, those with juvenile macular dystrophy did not overestimate the contrast of higher spatial frequencies using their PRL. This represents a degree of contrast constancy at the PRL, which is a phenomenon normally characteristic of the normal fovea to help discriminate suprathreshold contrast more accurately than in the periphery. In normal peripheral retina, contrast overconstancy occurs, creating a tendency for the observer to overestimate the contrast of higher spatial frequencies, or more detailed features, in the periphery (Mei & Leat 2007a).

Crowding refers to the reduced ability to identify and discriminate, rather than detect targets, particularly in the peripheral field, when surrounded by other targets (Whitney & Levi 2011). For example, discriminating a letter within a string of other letters. Of the two studies measuring crowding, Casco et al. (2003) found that crowded visual acuity at 2.5° retinal eccentricity in an observer with HMD, with a 10° absolute scotoma, was not significantly different to that of controls. Results were reported in terms of z scores. Here z = 0.19, with 1.65 indicating statistical significance. At the PRL of three participants, compared to normal eccentric retina, Chung (2013) demonstrated significantly smaller radial critical spacing and radial-tangential anisotropy, resembling that of the normal fovea. Anisotropy indices were calculated from the critical spacing of the radial and tangential axes. Subsequently, t-tests were performed on the anisotropy indices of the PRL and normal peripheral retina of controls, revealing a significant difference of p < 0.0001. Therefore, in order to successfully discriminate a string of letters presented along a radial axis at the PRL, smaller (critical) spacing between letters is needed compared to normal eccentric retina. Also, a similar amount of space between letters is required for discrimination along both a radial or tangential axis at the PRL, whereas typically less space is needed along a tangential axis in normal eccentric retina.

Contour integration is an important step in object recognition and describes the grouping of local elements to form outlines of shapes (Loffler 2008; Ya, Yonghui & Sheng 2019). Nugent et al. (2003) did not state the specific statistical tests used or all p-values clearly but reported no significant difference in contour integration at the PRL of an observer with juvenile macular dystrophy or corresponding peripheral retina of controls.

Global form, global motion and biological motion were investigated by Burton et al. (2016) at 10° eccentricity. Global form is the processing of features to obtain the shapes of objects, leading to object recognition (Chung & Khuu 2014). Whilst global motion is the processing of features to ascertain the movements of objects (Furlan & Smith 2016). Biological motion is specific to the motion of living organisms, such as people and plays a crucial role in social behaviour (Pavlova 2012). As with Mei and Leat (2007), contrast sensitivity data was Log-transformed in order to perform parametric statistics. Repeated-measures analysis of variance then revealed global form and global motion performance to be significantly impaired at all light levels in achromats compared to controls (p < 0.001 and p = 0.001, respectively). However, biological motion performance was not (p = 0.139, the statistical significance limit was not specified). Further analysis found that this may be due to the significantly superior performance in two of three of the achromats and comparable performance of the other to controls under scotopic conditions. All three of these participants also demonstrated comparable scotopic contrast sensitivity and global motion to controls (Burton et al. 2016).

A lexical decision test was conducted by Casco et al. (2003). Lexical decision tests how well responders can distinguish words from non-words (Meyer & Schvaneveldt 1971). Results were reported in terms of z scores initially. A single HMD observer was found to perform significantly better than controls when identifying words from non-words at 5° eccentricity, bordering the scotoma (z = +2.6, with 1.65 indicating statistical significance). Similar to their results for crowded visual acuity, at 2.5° eccentricity, within the scotoma, results were non-significant, with controls performing slightly better (z = –1.6). The chi-squared test was also used to compare the effect of eccentricity on results in both groups, which showed that task performance was significantly better in the HMD observer than controls at 5° compared to 2.5° (p < 0.01) (Casco et al. 2003).

Casco et al. (2003) also conducted a simple visual search task. Visual search tasks involve detecting the presence of a target amongst other stimuli, known as distractors or clutter. The visual search task for this study was performed with differing amounts of clutter. No significant differences in performance were observed when clutter sizes were large or medium; however, for the smallest set size, the HMD observer performed better than controls, for which results just reached statistical significance. Results were reported in terms of z scores with +1.65 indicating statistical significance. The z-scores were as follows: +0.11, –0.35 and +1.65, for large, medium, and small set sizes respectively. Sensitivity to target detection, as measured in signal detection theory, was additionally calculated, adding to statistical significance data by providing a measure of accuracy and discriminability independent from bias. This helps in determining factors associated with better performance and decision thresholds (Phillips et al. 2001; Swets & Pickett 1982). A higher d’ value compared to controls was found for the HMD observer in the smallest set size (approximately d’ = 3.75 vs. 2.2), similar values for the medium set size (approximately d’ = 2 for both groups), but a lower d’ than controls for the largest (approximately d’ = 1.8 vs. 2.5).

Finally, implicit and explicit object recognition at large retinal eccentricities (30° and 50°) were studied in HMD observers and controls (Boucart et al. 2010). Explicit recognition refers to the conscious discrimination of objects by the visual system (Bar et al. 2001), whilst implicit recognition is the non-conscious discrimination of objects through repeated exposure and memory (Tallon-Baudry & Bertrand 1999). HMD observers were only tested at 50° due to the large scotoma sizes in this sample which exceeded 30°. The authors were concerned that testing within the scotomatous region would impede performance on the task; however, this may not have been the case, as shown by Casco et al. (2003), where superior performance was found on some tasks performed within the scotoma area. Analysis of variance was performed on results and signal detection theory applied. Although not all p-values were reported, the authors report that at 50°, HMD observers had a similar pattern of results to controls but performed less accurately overall on explicit and implicit object recognition tasks. Both groups had low d’ values on the explicit object recognition task (d’ ≤ 0.225) indicating that performance was near chance and sensitivity was very low (Boucart et al. 2010), reflecting a possible floor effect.

## Discussion

This systematic review highlighted the paucity of literature in this field. Studies found often lacked validity due to small heterogeneous samples and deficiencies in reporting of methods and population characteristics. Additionally, a range of peripheral visual abilities were tested at different eccentricities across samples. Therefore, direct comparisons of results were not always possible and conclusions could not be made with confidence.

It is important to note that as a literature review, there is a risk of bias if relevant studies were missed. In order to minimise this source of bias, a clear methodological approach was taken to the literature search including searching large databases and using strict eligibility criteria.

Nonetheless, regardless of retinal eccentricity, HMD observers demonstrated impairments of peripheral contrast sensitivity in brighter conditions but less impairment at lower spatial frequencies or in scotopic conditions. Impaired contrast sensitivity observed in brighter conditions and with higher spatial frequencies likely reflects the dysfunctional cones, which limit photopic perception and perception of detailed features. Comparable contrast sensitivity to controls at lower spatial frequencies or in scotopic conditions indicates an intact normally functioning rod system, which operates at lower spatial frequencies and under low light levels, as it would in a healthy retina (Burton et al. 2016).

Impairment in lower spatial frequency or scotopic contrast sensitivity observed in HMD, particularly achromats, may indicate additional rod impairment within this population, where some patients exhibit atypical development of rods or reduction in rod sensitivity over time (Khan et al. 2007; Nishiguchi et al. 2005). This could represent part of the natural history of this disease, as has been shown in rod-cone dystrophies, such as retinitis pigmentosa, where despite the genetic mutation being specific to rod photoreceptors, cones become affected over time (Ripps 2002).

Where low spatial frequency information is maintained, unlike form perception, motion perception may also be comparable or even superior to controls. This may reflect the significance of cones in form perception, whilst rods play a greater role in motion perception (Lee et al. 1997; Maunsell, Nealey & DePriest 1990; Sun, Pokorny & Smith 2001; Wilkinson et al. 2000; Wilson, Wilkinson & Asaad 1997; Wilson & Wilkinson, 1998). Impaired form perception may explain why HMD observers performed worse in an object recognition task at 50°. Low performance was observed in the explicit recognition task for both groups compared to the implicit recognition task at this eccentricity. This demonstrates a greater reliance on non-conscious perceptual identification through repetition and memory, rather than the conscious discriminating power of the visual system at this eccentricity, which may reflect a limit of perception at 50° (Boucart et al. 2010). Those with larger scotoma may therefore be limited in their capacity to develop peripheral visual abilities but may benefit from repeated exposure to objects and environments through perceptual learning.

Interestingly, contour integration, a building block of form perception (Loffler 2008) and object recognition (Ya, Yonghui & Sheng 2017), was found to be similar to controls (Nugent et al. 2003). Perhaps performance was not worse on this task as participants specifically used their PRL, which has been developed to act as a foveal replacement. However, it is important to remember that the sample size was very small, reducing reliability of results.

The PRL has, however, been found to have more similar functional properties to the normal fovea than peripheral retina, improving performance on peripheral tasks, despite limitations from significantly reduced cone density. Properties include contrast constancy and lack of radial-tangential anisotropy, demonstrating some compensation for deficits in peripheral contrast perception and crowding at the PRL. This may aid form perception (Chung 2013; Mei & Leat 2007a), which is necessary for reading (Cui et al. 2019) and object recognition (Loffler 2008), including face recognition (Chung & Khuu 2014; Tsao & Livingstone 2008).

Where visual search is required under less cluttered conditions, HMD observers may perform better than controls. However, increasing task difficulty, in terms of clutter elements, appears to reduce performance differences between the two groups (Casco et al. 2003). The limitations of the peripheral field may not have allowed for this superior performance under more cluttered conditions, but may have if the PRL was used, considering the similarities with a normal fovea mentioned above.

Variability in results across studies could also be affected by variation in severity of central retinal involvement in these study populations, as it is theorised that denser central scotoma should manifest greater compensatory changes to vision (Dilks et al. 2014). However, without larger samples and adequate recording of participant visual characteristics it is not possible to investigate this further.

Perceptual learning may be an explanation for superior performance of some tasks in HMD observers (Casco et al. 2003; Cheung & Legge 2005; Plank et al. 2013; 2014). These authors proposed that those with early onset central field loss have spontaneously trained their peripheral retina and PRL in particular, to maximise use of information with low spatial resolution to perform central-vision related tasks essential in daily life. For example, peripheral lexical decision and crowding may be improved through reading; peripheral visual search may be improved through searching for objects, words, and people in the real world, on screens or paper; and peripheral biological motion processing through daily social interaction. Alternatively, Chung (2013) did not consider tasks of daily living sufficient for perceptual learning to take place, as intensive laboratory training is typically necessary (Fahle, 2005; 2008; Fiorentini & Berardi 1980; Gilbert, Sigman & Crist 2001; Li, Piech & Gilbert 2004; Tsodyks & Gilbert 2004). Chung (2013) instead suggested cortical reorganisation of V1 as the primary explanation for superior performance in their juvenile-onset macular dystrophy observers. However, neurophysiological studies do not support cortical reorganisation in juvenile-onset macular dystrophy (Baseler et al. 2011; Liu et al. 2010; Masuda et al. 2008; Plank et al. 2017), but they do for functional reorganisation, which can be induced by perceptual learning.

The evidence from this review for spontaneous perceptual learning resulting in some superior peripheral abilities in HMD observers compared to controls and comparable abilities at the PRL and normal fovea, encourages the promotion of further active rehabilitation to maximise these abilities. Moreover, where abilities have been found to be inadequate, exposure from daily tasks may be insufficient for spontaneous perceptual learning and active rehabilitation may be necessary.

Tasks demonstrating superior peripheral abilities in this review highlight tasks which may be most useful in rehabilitative training regimens as they are clearly important enough to HMD observers’ daily lives to have been developed. Larger, more rigorous studies are needed to establish the superior peripheral abilities found in this review and to identify superior performance in other peripheral visual tasks which may reflect the visual abilities most needed for the daily functioning of HMD observers that can be trained. These tasks can then be incorporated into effective training regimens. Likewise, further research is also needed to identify deficient abilities which may be useful but require active rehabilitation.

Training regimens have been shown to improve task performance in deficient abilities with transferability to daily tasks. A systematic review concluded that, despite the low quality of evidence available, regardless of the model of eccentric viewing training or steady eye strategy, near visual acuity, reading speed, and daily task performance can be improved through these simple training tasks (Gaffney et al. 2014). Despite these positive findings, the research is still in its infancy and there is currently not enough high-quality evidence, including randomised controlled trials, to produce guidelines on the best training methods or most cost-effective training regime.

Furthermore, this review shows that it is indeed intuitive to focus training at the PRL, as the PRL has been found to mimic foveal functioning somewhat (Chung 2013; Mei & Leat 2007a). However, this review also shows that improvements may be possible for the remaining peripheral field as a whole; therefore, training should not only be focussed at the PRL, which could become affected through disease.

The results of this review calls for further research, which can affect clinical management of patients with HMD. As some HMD observers were found to have some specific enhanced peripheral visual abilities, the investigation of other abilities such as visual field size that has been found to expand in action video game players and the congenitally deaf, is warranted. These results may have clinical implications for visual field interpretation if found to be enhanced also. Finding a typically normal peripheral visual field in patients with HMD could be indicative of pathology, which may currently be misinterpreted as normal. On the other hand, as mentioned previously, those with HMD may show impairment in the periphery, possibly due to rod involvement over time. This is an important prognostic factor which should be discussed with patients when counselling them about their condition. In addition to other clinical tests, it should also be encouraged to perform perimetry regularly, to monitor the peripheral field. This will aid the prompt detection of peripheral impairment and early rehabilitation, from a mobility, emotional, and psychosocial perspective.

## Conclusions

Spontaneous perceptual learning through reliance on and repeated use of the peripheral field and PRL may result in some superior peripheral visual abilities in early onset central field loss. However, worse performance in some tasks could reflect rod disease, lack of intensive training, or persistent limitations due to the need for cones for specific tasks. To facilitate further improvements, or where abilities are inadequate, perceptual learning through training regimes could optimise use of the PRL and remaining peripheral vision.

There is a need to repeat the current studies in this review with larger samples and more rigorous methods to increase the validity of findings. Future studies should also investigate other peripheral visual abilities to broaden knowledge of behavioural and neural adaptations of those with early onset central field loss and to identify abilities which require rehabilitation to maximise visual potential and performance for daily tasks. Finally, further studies are needed to enable the design of optimal training regimes.

## Appendix 1

**Table 1:** Summary of included studies

	STUDY	STUDY DESIGN	SAMPLE	MEASUREMENT(S)	MAIN FINDINGS/CONCLUSIONS

1	Boucart et al (2010)	Case-control	SMD with 10–20 years of central field loss (n=4) (age range 26–31) Normally-sighted controls (n=15) (age range 24–40) Gender not reported.	Explicit and implicit object recognition at 30° and 50° retinal eccentricity for controls and 50° for SMD.	Controls and SMD had a similar pattern of results with SMD performing slightly less accurately. No evidence for cortical plasticity.

2	Burton et al (2016)	Case-control	Achromatopsia (n=11) (age range 19–50) (6 M/ 5 F) Normally-sighted controls (n=20) (age range 18–38) (11 M/ 9 F)	Contrast sensitivity function, global form, motion and biological motion detection at 10° eccentricity. All tasks at 4 different light levels.	Greater impairment of global form and motion in all light levels in achromatopsia compared to controls. But, comparable or in some, superior biological motion processing in scotopic conditions in achromatopsia. Contrast sensitivity was variable but generally worse than controls. Some evidence in support of cortical plasticity from reliance on rod-vision.

3	Casco et al (2003)	Case-control	SMD (n=1) (age 21) (F) Normally-sighted controls (n=8) (states age-matched, but age range not stated)	Contrast sensitivity function at 10° eccentricity. Lexical decision at 2.5° and 5° eccentricity. Visual acuity at 2.5° eccentricity. Visual search at 10° eccentricity or greater (not specified).	In SMD, contrast sensitivity impaired, but not for low spatial frequencies. Better performance on peripheral lexical decision in SMD. Similar visual acuity between groups. Better performance on simple visual search in SMD. Evidence for cortical plasticity that produces improvements through perceptual learning from long-term training in lexical decision and visual search in daily life.

4	Chung (2013)	Case-control	AMD (n=8) (age range 73–85) (3 M/ 5 F) SMD (n=3) (age range 48–62) (2 M/ 1 F) Normally-sighted controls (n=8) (age range 63–79) (3 M/ 5 F)	Radial and tangential critical spacing on crowding task using PRL in SMD/AMD, but set eccentric locations in controls.	Radial critical spacing shrinks, reducing radial-tangential anisotropy at the PRL of AMD/SMD, resembling foveal rather than peripheral crowding functions. Evidence for cortical plasticity.

5	Hess, Nordby and Pointer (1987)	Case-control	Achromatopsia (n=1) (co-author but age not stated) Normally-sighted controls (n=2) (includes co-author but ages not stated) Gender not reported.	Contrast threshold at 0- 25° eccentricities. All tasks at 5 different light levels.	In scotopic light, spatio-temporal contrast sensitivity of the achromat and controls are similar. No evidence for cortical plasticity.

6	Mei and Leat (2007)	Case-control	Atrophic AMD (n=13) (age 81±5, no rang given) Exudative AMD (n=14) (age 81±8, no range given) JMD (n=8) (age 47±14, no range given) Normally-sighted controls (n=15) (age 70±11, no range given) Gender not reported.	Contrast threshold and contrast matching with free use of PRL for non-controls and fovea for controls.	Impaired contrast sensitivity thresholds for AMD/JMD but not for low spatial frequencies. Suprathreshold contrast constancy was evident at the PRL in AMD/JMD resembling foveal rather than peripheral functioning, but with deficits compared to controls. Some evidence for cortical plasticity

7	Nugent et al (2003)	Case-control	JMD (n=1) (age range 47–58) Also 2 other participants, one with an unclear diagnosis and the other with peripheral field involvement. Normally-sighted controls (n=5) age range 20–33) Gender not reported.	Contour integration with free use of PRL for JMD and at 0-30° eccentricities for controls.	Similar performance between single JMD participant and controls for detecting contours. No evidence for cortical plasticity.

Abbreviations: Stargardt’smacular dystrophy (SMD), Age-related macular degeneration (AMD), Juvenile maculardystrophy (JMD), Preferred retinal locus (PRL), Male (M), Female (F).
